# Limiting the Use of Electromyography and Ground Reaction Force Data Changes the Magnitude and Ranking of Modelled Anterior Cruciate Ligament Forces

**DOI:** 10.3390/bioengineering10030369

**Published:** 2023-03-17

**Authors:** Azadeh Nasseri, Riad Akhundov, Adam L. Bryant, David G. Lloyd, David J. Saxby

**Affiliations:** 1 Griffith Centre of Biomedical and Rehabilitation Engineering (GCORE), Menzies Health Institute Queensland, Griffith University, Southport, QLD 4222, Australia; 2 Centre for Health, Exercise & Sports Medicine, University of Melbourne, Melbourne, VIC 3010, Australia

**Keywords:** vertical and 3D ground reaction force, electromyography, static optimization

## Abstract

Neuromusculoskeletal models often require three-dimensional (3D) body motions, ground reaction forces (GRF), and electromyography (EMG) as input data. Acquiring these data in real-world settings is challenging, with barriers such as the cost of instruments, setup time, and operator skills to correctly acquire and interpret data. This study investigated the consequences of limiting EMG and GRF data on modelled anterior cruciate ligament (ACL) forces during a drop–land–jump task in late-/post-pubertal females. We compared ACL forces generated by a reference model (i.e., EMG-informed neural mode combined with 3D GRF) to those generated by an EMG-informed with only vertical GRF, static optimisation with 3D GRF, and static optimisation with only vertical GRF. Results indicated ACL force magnitude during landing (when ACL injury typically occurs) was significantly overestimated if only vertical GRF were used for either EMG-informed or static optimisation neural modes. If 3D GRF were used in combination with static optimisation, ACL force was marginally overestimated compared to the reference model. None of the alternative models maintained rank order of ACL loading magnitudes generated by the reference model. Finally, we observed substantial variability across the study sample in response to limiting EMG and GRF data, indicating need for methods incorporating subject-specific measures of muscle activation patterns and external loading when modelling ACL loading during dynamic motor tasks.

## 1. Introduction

Rupture of the anterior cruciate ligament (ACL) is a debilitating knee injury, associated with an elevated risk of subsequent early-onset knee osteoarthritis [1]. Over the past two decades, rates of ACL rupture have increased alarmingly, with young females showing greatest yearly increases [2]. Recent advances in computational modelling of the ACL enable accurate quantification of ACL loads during dynamic tasks performed in laboratory settings [3,4]. If this research technology could be made “field-friendly”, this would enable assessment of ACL loading in ecologically valid settings (e.g., training fields, gymnasiums, clinics). Successful commercialisation of a field-friendly “ACL Force Calculator” (#PCT/AU2019/051129) would empower clinicians, coaches, teachers, and parents to develop interventions directly targeting personalised mechanisms of ACL loading, thereby reducing risk of ACL rupture.

The abovementioned computational model of the ACL uses the performer’s three-dimensional (3D) body motion, body-environment reaction loads (e.g., ground reaction forces and moments; GRF), and muscle activation patterns (i.e., electromyograms; EMG) to drive a neuromusculoskeletal model that quantifies ACL force [3,4]. However, acquiring 3D body motion, GRF, and EMG in real-world settings (e.g., community sports fields, school gymnasiums, or physical therapy clinics) is challenging with barriers such as instrument costs, setup time, and operator skills to acquire and interpret data correctly [5]. Wearable sensors (e.g., inertial measurement units) and marker-free optical systems (using sophisticated human body models, computer vision, and machine learning algorithms) have been suggested as alternatives to traditional laboratory-based optical motion capture. Indeed, these alternatives can accurately estimate landmarks and body segment motions [6,7]. However, the validity of these methods has not been demonstrated across different movements and environments [8]. Moreover, wearable devices might be impractical in some instances due to cost or posing a hazard to the wearer, others, or the device (e.g., contact sports). If it were shown that GRF and EMG could be simplified, or altogether avoided, while maintaining accuracy of predicted ACL forces, this would be of substantial practical value to translating methods to estimate ACL force from the research laboratory to the real-world.

Wearable accelerometers in combination with deep learning models [9] or depth cameras in combination with musculoskeletal models [10] might facilitate in-field prediction of multidimensional GRF. Wearable technologies to estimate vertical GRF in-field exist and use physics-based [11] and artificial intelligence (AI) approaches [12,13,14], although their ability to estimate GRF for different tasks and environments is yet to be verified. Thus, the methods hold promise, but generalisability across tasks has not been established. Hence some researchers have focused primarily on the vertical component of GRF, reasoning its large magnitude compared to other components during human locomotion renders it most important biomechanically [15,16,17,18]. One-dimensional (vertical) GRF instruments (e.g., ForceDecks; VALD Performance, Queensland, Australia) can be used in-field, and traditional in-shoe pressure insoles are well accepted in clinical gait laboratories. Wearables (combined with computational methods) and simplified GRF instruments are inexpensive compared to three-dimensional (3D) force platforms and more portable, thereby making them potentially viable assets in the quest for in-field modelling of ACL loading.

Experimental measures of muscle activation (i.e., EMG) can be used to inform computational models of muscle dynamics [19], providing a subject- and task-specific approach to the problem of load sharing within the body. However, the inclusion of EMG into a field technology adds cost and complexity, potentially making such technology commercially unviable. Recent research indicates the activation patterns of many of the lower limb muscles could be estimated through a synergy extrapolation method using only a small number of experimentally measured EMG to predict a larger set [20]. However, muscle synergy approaches appear to be task-specific, limiting their utility for in-field technology where many different motor tasks might be assessed. In lieu of EMG or synergy methods, optimisation methods have been used since the 1970s to solve the muscle redundancy problem [21]. Static optimisation is the most commonly used method, as it is computationally advantageous compared to other optimisation approaches (e.g., computed muscle control, direct colocation) [22]. Nuances in the specific implementation exist (e.g., stiff vs elastic tendons, efficiencies in gradient formulation) [23,24], however, static optimisation generally functions by minimising activation (or stresses) of muscles such they satisfy the joint moment profile of the task studied [21,25,26]. Although static optimisation was inspired by a physiological criterion [27,28,29], more recent research has shown muscle activation to be specific to both individual and control tasks [30,31], as well as being sensitive to training history [32] and pathology [33]. Simply put, static optimisation does not mathematically represent the complexity of human muscle recruitment, although what effect this limitation has on modelling ACL loading is unclear.

This study aimed to understand the consequences of limiting the full use of EMG and GRF on the magnitude and rank order of the model-predicted ACL loads. Past literature indicates joint contact forces are sensitive to the neural mode used to estimate muscle forces, where, for example, static optimisation overestimates muscle and knee joint contact forces compared to EMG-informed models [34]. Therefore, we hypothesised static optimisation would consistently overestimate ACL force. Furthermore, we hypothesised limiting 3D GRF to only the vertical component would increase ACL force as it eliminates the braking reaction force applied to the body during the task and minimises non-sagittal knee loading. Finally, we hypothesised the rank order of participants based on the magnitude of their ACL forces would be different between modelling configurations, indicating the sensitivity of ACL loading to subject-specific muscle activation and external loading patterns. To achieve the aim of the study, we perform a secondary analysis of a previously modelled drop–land–jump task [35]. We assumed an EMG-informed neural mode in combination with three-dimensional (3D) GRF as a reference configuration. Against this reference, we compare ACL forces modelled using EMG-informed with only vertical GRF, static optimisation with 3D GRF, and static optimisation with only vertical GRF.

## 2. Methods

### 2.1. Participants

In this study, we used data acquired from 23 healthy late-/post-pubertal females who were a subset of participants from a larger study conducted by the University of Melbourne’s Centre for Exercise, Health & Sports Medicine (ethical approval #1442604) [36]. Those analysed in the present study had a mean (±standard deviation) age, mass, and height of 19.7 (±4.0) years, 59.7 (±9.5) kg, and 1.65 (±0.06) m, respectively. Participants had no history of lower-limb injury, knee pain, or ACL rupture. Participants aged over 18 years, or parents/guardians of those less than 18 years of age, provided their written informed consent.

### 2.2. Motor Task

Biomechanical measurements were performed in a laboratory setting. Each participant was asked to complete three trials of standardised motor tasks, including straight running and drop–land–jumping. The drop–land–jump task was selected as a controlled version of single-leg landing and sidestepped manoeuvre performed in many sports, which provokes ACL loading. The participant stood barefoot on their dominant leg, dropped off an elevated box onto a marked point on the ground, and immediately jumped to their contralateral side to land on their other (non-dominant) leg. The task was standardised across participants by adjusting the box height to 30% of leg length, positioning the box 10 cm back from the marked centre of the force plate, and ensuring the lateral jump was performed to 150% of leg length. Standardisation had the effect of normalising starting height across participants [3]. Running trials were only used for neuromusculoskeletal model calibration (explained later) and were not subject to analysis.

### 2.3. Measurements

Participants were outfitted with retroreflective markers placed on their feet, shanks, thighs, pelvis, and trunk. A 12-camera Vicon (Oxford Metrics, Oxford, UK) motion capture system acquired marker positions at 120 Hz. Muscle activation patterns were acquired by placing Ag/AgCl electrodes atop eight major knee-spanning muscles (i.e., rectus femoris, vastus lateralis, vastus medialis, tibialis anterior, lateral gastrocnemius, medial gastrocnemius, lateral hamstrings, and medial hamstrings) of the dominant limb, consistent with SENIAM guidelines [37], while a wireless system (Noraxon, AZ, USA) acquired EMG signals at 2400 Hz. To orient the participant during jumping, the marked target on the ground was placed atop a force platform (AMTI, MA, USA) which acquired 3D GRF at 2400 Hz. For this study, we used both 3D GRF and only vertical GRF in subsequent modelling configurations (explained below).

### 2.4. Signal Processing

Retroreflective marker positions were first labelled, and then small gaps (<10 frames) were interpolated using Vicon Nexus software. Data were then exported and elaborated in MATLAB (version 2019a, MathWorks, MA, USA) using the motion to neuromusculoskeletal modelling toolbox (MOtoNMS) [38]. Marker positions and GRF were filtered using a second-order Butterworth low-pass filter, cascaded once to remove phase effects, with a nominal cut-off of 6 Hz. The EMG were first band-pass filtered (30–300 Hz), full-wave rectified, and low-pass filtered (6 Hz cut-off) to yield linear envelopes, and then the individual amplitude normalised using corresponding maximum envelope values from all available trials [39]. The EMG signals and their corresponding linear envelopes were then assessed for EMG quality and appropriateness for neuromusculoskeletal modelling using an EMG classification tool [40].

### 2.5. Neuromusculoskeletal Modelling

To quantify internal tissue biomechanics (e.g., ACL forces), we followed a conventional modelling pathway. This involved first adapting a generic anatomical model to each participant, followed by modelling the external biomechanics (e.g., generalised coordinates and loads), both performed in OpenSim [26]. The next step involved establishing four neuromuscular modelling configurations (EMG-informed and static optimisation, each with 3D and only vertical GRF). Finally, external biomechanics and neuromuscular outputs were used in a validated phenomenological model of ACL loading [41].

The generic anatomical model used in this study was a modified version of that presented by Rajagopal and colleagues [42]. This full-body model provided 37 degrees of freedom, spanned by 80 muscle-tendon unit (MTU) actuators. The knee mechanism of this generic model was modified by adding dummy bodies of negligible mass and inertia [43]. These bodies were non-contributing to model kinematics as they had zero mobility space, but enabled calculation of generalised loads about 6 degrees of freedom at the knee, which is not otherwise possible using the model of Rajagopal and colleagues [43].

The modified anatomical model was then linearly scaled to match the body segment dimensions, mass, and inertia of each participant. We then performed morphometric scaling to ensure dimensionless muscle and tendon operating ranges were preserved after linear scaling [44]. Finally, we adjusted maximal isometric force of each muscle based on participant height and mass using equations developed by Handsfield et al. [45]. The scaled and muscle parameter-adjusted model was then used to determine external biomechanics in OpenSim. Model motion, MTU kinematics (i.e., instantaneous lengths, moment arms, and lines of action), and generalised loads (i.e., net joint forces and moments) were determined through inverse kinematics, muscle and line of action analyses [46], and inverse dynamics, respectively. Muscle and joint contact force estimates were implemented using the calibrated EMG-informed neuromusculoskeletal modelling toolbox (CEINMS) [19]. In CEINMS, MTU kinematics and muscle activations are used as inputs for a Hill-type muscle model with a compliant tendon.

For each participant, all drop–land–jump trials were modelled in CEINMS using static optimisation or EMG-informed muscle force estimation, each with full 3D GRF and only vertical GRF. The combinations are referred to as follows: static optimisation + 3D GRF; EMG-informed + 3D GRF; static optimisation + vertical GRF; and EMG-informed + vertical GRF. In CEINMS, static optimisation synthesises all muscle excitations for MTU with an elastic tendon. Optimal muscle activations were found by minimising sum of squared activations, which generate lower-limb flexion/extension moments and function under the assumption that the central nervous system favours a neural solution minimising metabolic cost [29]. The EMG-informed neural mode used experimental EMG signals and synthesised muscle activations to estimate lower limb joint moments. This approach was formulated as an optimisation problem,

(1) $$E=\propto E_{\tau }+\gamma E_{emg}+\beta E_{ex}$$

where, an optimised excitation set is found by minimising error term, E, which is composed of sum of squared errors between model-generated and inverse dynamics moments, $E_{\tau }$, sum of squared errors between recorded EMG and synthesised excitations, $E_{emg}$, and summed excitations of all MTU involved, $E_{ex}$. Weighting terms ∝, γ, β are positive, with ∝ and β set to 1, while γ is optimised to minimise deviation from measured EMG.

For both static optimisation and EMG-informed approaches, calibration was performed once for each participant, followed by execution for each trial. One straight running and one drop–land–jump trial were used for calibration. Calibration is an optimisation process whereby design variables (i.e., optimal muscle fibre and tendon slack lengths, excitation and activation dynamics, and maximal isometric muscle force) are adjusted (within physiological constraints) to minimise error between model-generated and inverse dynamics joint moments (e.g., flexion and extension moments about hip, knee, and ankle) [19]. Once calibrated, CEINMS was used in static optimisation and EMG-informed modes to compute muscle dynamics for each drop–land–jump trial.

### 2.6. Anterior Cruciate Ligament Force Modelling

To estimate ACL force, a validated phenomenological model was used [41]. This model requires knee-spanning muscle forces and their lines of action, knee posture, and generalised knee loads as inputs. Output provides tension (N) developed in ACL and contributions (N and %) from loads developed about different knee planes of motion and from all knee-spanning muscles.

### 2.7. Statical Analyses

To assess consequences of limiting EMG and GRF on modelled ACL forces, time-varying and discrete statistical analyses were performed. First, ACL force time–series during stance phase of drop–land–jump were compared between the four modelling configurations using a one-way repeated measures ANOVA deployed through statistical parametric mapping (SPM) [47]. When a statistically significant SPM ANOVA effect of model configuration was found, post hoc comparisons amongst modelling approaches were undertaken using SPM *t*-tests with a Bonferroni correction. A conservative alpha of 0.008 was used to determine statistical significance, due to the six comparisons made. Second, ACL force time-series across stance phase of drop–land–jump was parameterised, and peaks were analysed. Third, participants were ranked by their peak ACL force for each of the four modelling approaches. Rank order was then compared between modelling approaches using Kendall’s rank correlation [48]. All statistical analyses were performed in MATLAB (version 2019a, MathWorks, MA, USA).

## 3. Results

**Figure 1:**
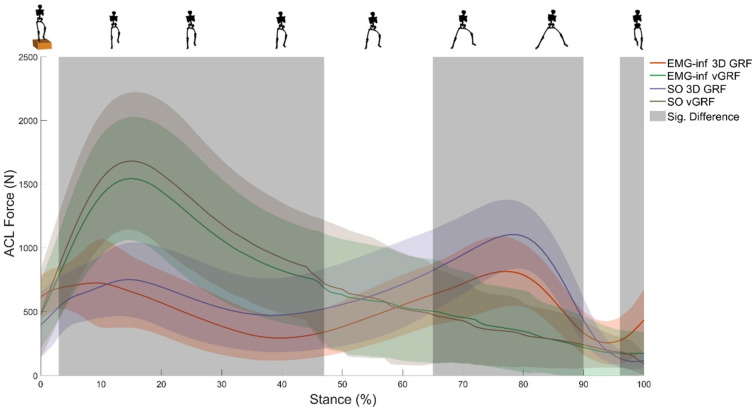
Ensemble average (±1 standard deviation) total ACL force over the stance phase of the barefoot drop–land–jump estimated via four modelling approaches: EMG-informed + 3D GRF (red line); Static optimisation + 3D GRF (purple line); EMG-informed + vertical GRF (green line); Static Optimisation + vertical GRF (brown line). The grey-shaded region displays the areas of significant statistical differences using one-way repeated measures ANOVA applied through statistical parametric mapping. vGRF, vertical ground reaction force; EMG-inf, EMG-informed; SO, static optimisation.

**Figure 2:**
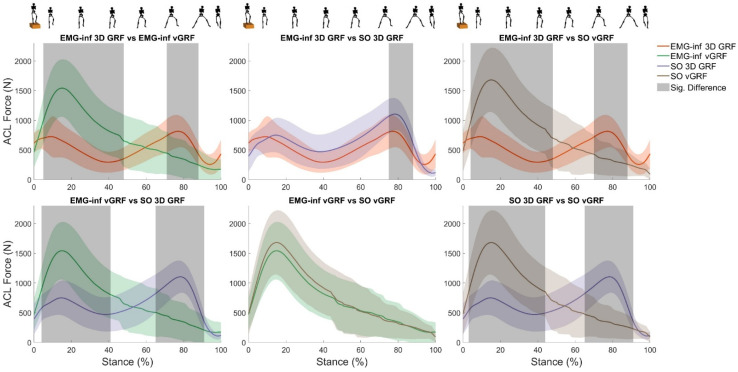
Pairwise comparisons of total ACL force (±1 standard deviation) over the stance of the barefoot drop–land–jump estimated via four modelling approaches: EMG-informed + 3D GRF (red line); Static optimisation + 3D GRF (purple line); EMG-informed + vertical GRF (green line); Static Optimisation + vertical GRF (brown line). Grey-shaded regions display statistical differences using a paired t-test applied through Statistical Parametric Mapping. vGRF, vertical ground reaction force; EMG-inf, EMG-informed; SO, static optimisation.

**Figure 3:**
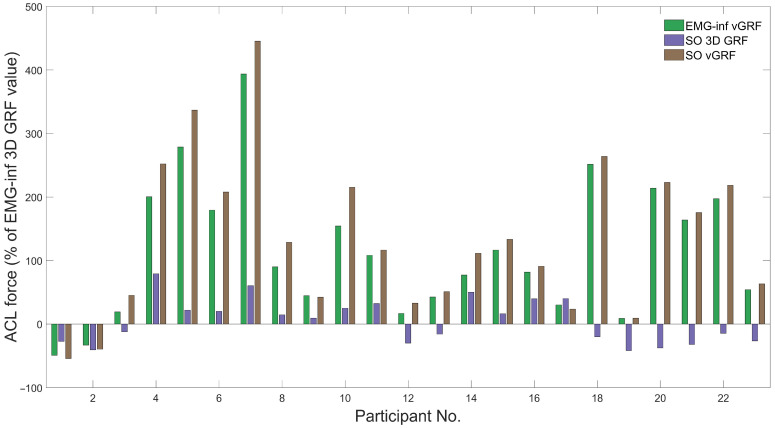
Relative differences (%) in peak ACL force during barefoot drop–land–jump between the EMG-informed + 3D GRF and other modelling approaches: EMG-informed + vertical GRF (green), static optimisation + 3D GRF (purple), and static optimisation + vertical GRF (brown). EMG-inf, EMG-informed; vGRF, vertical ground reaction force; SO, static optimisation.

The SPM ANOVA revealed significant main effects of model configuration on ACL force for most of stance (Figure 1). *Post hoc* analysis revealed significant differences in ACL force magnitude between all model configurations (Figure 2), except for the comparison between EMG-informed + vertical GRF and static optimisation + vertical GRF. Compared to EMG-informed + 3D GRF, both EMG-informed and static optimisation methods using only vertical GRF generated significantly higher ACL forces (mean differences, 205.5 N and 253.8 N, respectively) for the majority of stance (Figure 2). When 3D GRF was used, differences between ACL force generated by EMG-informed and static optimisation neural modes were observed for a small duration within the final 20% of stance (mean differences, 116.4 N, (Figure 2). Compared with static optimisation + 3D GRF, both EMG-informed + and static optimisation + vertical GRF generated significantly higher ACL forces (mean differences, 89.1 N and 137.4 N, respectively) for most of stance (Figure 2). For 87% of participants, modelling ACL forces using only vertical GRF generated, independent of different neural mode (e.g., EMG-informed or static optimisation), the largest differences in peak ACL force magnitudes compared to EMG-informed + 3D GRF (Figure 3).

Based on their peak ACL force, rank order of participants was compared across modelling approaches (Table 1). No statistically significant correlations in rank order were found between EMG-informed + 3D GRF and the other three configurations (Table 2).

## 4. Discussion

We examined the consequences of limiting EMG (i.e., via static optimisation) and GRF (i.e., using only the vertical component) on model-estimated ACL force generated during a standardised and controlled motor task that provoked ACL loading. Compared to using 3D GRF, using only the vertical component of GRF with either EMG-informed or static optimisation neural modes, resulted in an overestimation of ACL force during the first half of stance, which is when ACL injury typically occurs. In contrast, when 3D GRF were used, static optimisation neural mode only marginally overestimated ACL forces compared to an EMG-informed model. The order of participants ranked based on their peak ACL forces was not preserved across model configurations. This means modelling choices affect not only the absolute magnitude of ACL forces but also relative ranking of an individual within a cohort. Overall, we found 3D GRF was more important to modelling ACL force in the drop-land-jump task than altering the neural mode, although simplifying the neural mode to static optimisation overestimated ACL loading even when modelled using 3D GRF.

Currently, vertical GRF can be measured in-field using AI [49,50], commercially available instrumented insoles [51], and one-dimensional force platforms [52]. This study demonstrated that using only vertical GRF resulted in significantly larger estimates of ACL loading (Figure 2, mean differences ranging from 89.1 to 253.8 N) compared to 3D GRF, irrespective of the neural model employed. This effect of limiting GRF on ACL loading was consistent with our hypothesis and unsurprising given sensitivity of both inverse dynamics and neuromusculoskeletal modelling to external loading and their complex mechanistic relationship with ACL loading (see Nasseri et al. for an in-depth explanation [3,4]). Although vertical GRF may be relevant for performance tracking [15,16,17,18] and is the largest component of the GRF signal during most human locomotion, our findings indicate its exclusive use resulted in inaccurate estimates of ACL loading during the drop–land–jump task. This is because the ACL force model is sensitive to the posteriorly directed external force at the knee (see Figure 2 in [4]). The drop–land–jump task in this study required participants to arrest their bodies’ forward momentum during landing and then immediately perform a lateral jump. Considerable posteriorly oriented inter-segmental forces are generated to produce the negative accelerations of the body’s segments (see [53] for commentary) during landing. To the best of our knowledge, no previous study has investigated the effects of limiting GRF on knee moments or ACL forces, making a direct comparison with the literature impossible. This finding of increased ACL force when 3D GRF is simplified to vertical GRF may not extend to different tasks that involve forward propulsion (rather than breaking) and might result in a different outcome, although the error would presumably be an underestimation of ACL force, rather than the overestimation, given the direction of anterior-posterior loads on the tibia. Furthermore, to minimise reliance on controlled research laboratory environments that use external force transducers, 3D GRF can be well estimated using AI if the 3D motion of selected body regions is measured reliably [49]. However, the validity of existing in-field measurements of 3D motion has yet to be fully established across different movements in varying environments [8].

Limiting the neural model from EMG-informed to static optimisation resulted in a larger ACL force, independent of using 3D or vertical GRF, consistent with our hypothesis. This overestimation of ACL force was statistically significant for only a small portion of stance. Previous studies indicate the incorporation of EMG into neuromusculoskeletal modelling increases accuracy of knee loading predictions [34,54]. At the same time, static optimisation requires additional constraints (i.e., lateral soft-tissue tensioning) to prevent non-physiological occurrences (e.g., lateral condyle unloading [55]) and often results in dramatic fluctuations in internal biomechanics (e.g., modelled ACL forces [56]). Prediction of non-physiological internal biomechanics may stem from static optimisation operating (as the name implies) quasi-statically, without influence from previous time point solutions nor accounting for observed phenomena of muscle coordination (e.g., force-sharing loops [57], antagonistic activation [58], and co-contraction [59]) that may limit rapid fluctuations in internal loading. Interestingly, the effects of reducing neural information from EMG-informed to static optimisation on ACL force magnitude were smaller than limiting 3D GRF to only the vertical component. This may reflect a sensitivity of ACL modelling to external task dynamics over muscle coordination in the task analysed here. We performed static optimisation through CEINMS using an elastic tendon, as opposed to the commonly used OpenSim static optimisation tool, which employs a rigid tendon. We believe using an elastic tendon within the static optimisation approach is a better method to estimate muscle forces when experimental EMG data are not available. Indeed, using inextensible tendon models produce large force errors as MTU length changes must be accounted for entirely through fibre kinematics [60].

Aligned with our hypothesis, limiting EMG or GRF data did not preserve rank order of peak ACL loading across participants (Table 1). This has implications for products designed to identify the highest loaded ACL from a cohort of participants (e.g., mass screening of candidate sports players or military recruits) using vertical GRF and optimised muscle activation patterns. Across participants in this study, effects of limiting EMG and GRF on ACL loading were inconsistent and highly variable. Indeed, one modelling configuration may have increased ACL loading for one participant, while resulted in decreased or unchanged ACL loading for other participants (Figure 3). We could find no obvious factor to discriminate amongst individual responses in ACL loading. Overall, highly variable and individualised responses to modelling configurations re-enforces the need to employ subject- and task-specific modelling methods that account for subject- and task-specific features such as muscle activation patterns.

There are several limitations that should be considered. First, we have deliberately ignored the issue of modelling 3D body motion using field-friendly technology and used laboratory-based motion capture for this study. We chose this approach as there are numerous studies using arrays of wearable sensors (e.g., XSens body suit), fusion of sensors and optical capture, and computer vision methods that have demonstrated their capability to model 3D motion accurately. We note the movements and environmental conditions tested are, to date, not exhaustive, but there is ample evidence that with an appropriately selected method one can compute 3D body motion in-field. Second, we assumed the combination of 3D GRF with EMG-informed NMS modelling and a validated ACL loading model as our gold standard to which the other three modelling configurations were compared. Since direct ACL force measurement was not possible in this cohort (or almost any healthy cohort), computational modelling of the human neuromusculoskeletal system and internal tissue biomechanics is the best available method to quantify ACL load. Moreover, the neuromusculoskeletal modelling approach we have used has been independently recognised for its excellence in modelling internal biomechanics (see [61]), and the ACL model has been validated across a wide range (in magnitude and complexity) of loading configurations. Third, our cohort was comprised exclusively of females, and as such, we cannot say if the results extend to males. We have no reason to doubt the extensibility of our results to males, as sex differences in neuromusculoskeletal biomechanics do exist but are generally small compared to the data reductions performed in this study (e.g., only vertical GRF). Fourth, the drop–land–jump was performed barefoot, while most athletic tasks are performed shod. Our previous study [35] did reveal footwear-specific effects on ACL loading, although these were confined primarily to the propulsive (secondary) phase of the drop-land-jump and not during landing, which is when ACL injuries usually occur [62]. Fifth, data were acquired from participants performing the various tasks in a laboratory setting. Results might be different if data were acquired during a field performance of the task, although there is no logical reason to think static optimisation and vertical GRF models would perform better or worse in a real-world setting than in the laboratory.

In conclusion, simplifying muscle activation patterns by using a static optimisation neural model rather than an EMG-informed model and reducing 3D GRF to only vertical GRF resulted in spurious model estimates of ACL loading. Compared to modelling with 3D GRF, using only vertical GRF (as could be well measured by in-field sports equipment or AI methods) resulted in much larger ACL forces due to a lack of a posteriorly directed GRF during landing. The consequence of neglecting EMG in favour of static optimisation was complex as activation patterns are subject-specific and thus highly variable, which resulted in a small but significant increase in ACL loading at group level. Finally, the rank order of participants based on their model-estimated peak ACL loading was not preserved across the model configurations used in this study. Findings indicate we should strive to include both EMG and 3D GRF when assessing ACL loading during dynamic tasks. Thus, we should focus on developing hardware and software solutions for accurate estimation of those valuable neuromusculoskeletal signals.

## Figures and Tables

**Table 1:** Ranking of participants based on their peak ACL force developed during drop–land–jump for EMG-informed + 3D GRF and other modelling approaches.

EMG-Informed 3D GRF	SO vGRF	EMG-Informed 3D GRF	SO 3D GRF	EMG-Informed 3D GRF	EMG-Informed vGRF
2	18	2	11	2	18
23	5	23	14	23	22
11	22	11	17	11	11
19	11	19	2	19	5
12	6	12	8	12	6
3	7	3	4	3	20
14	20	14	23	14	7
17	23	17	16	17	23
13	14	13	6	13	14
1	8	1	9	1	21
8	4	8	3	8	8
9	21	9	15	9	4
22	15	22	13	22	15
6	10	6	5	6	2
18	3	18	12	18	13
15	13	15	22	15	10
16	12	16	1	16	17
20	16	20	7	20	9
21	2	21	10	21	16
5	9	5	19	5	12
4	19	4	18	4	19
10	17	10	21	10	3
7	1	7	20	7	1
0×	=Identical Rank	0×	=Identical Rank	2×	=Identical Rank
					
3×	=Rank ± 1	0×	=Rank ± 1	0×	=Rank ± 1
					

SO, static optimisation; vGRF, vertical ground reaction force. Identical Rank, instances where the same participant had the same relative rank order of peak ACL force; Rank ± 1, the individual’s peak ACL force differs by one rank between modelling configurations.

**Table 2:** Kendall’s rank correlation of peak ACL force between EMG-informed + 3D GRF and each different modelling approach for the drop–land–jump task.

	SO + vGRF	SO + 3D GRF	EMG-Inf + vGRF
Correlation:	−0.028	−0.012	0.067
*p*-value:	0.876	0.958	0.676

SO, static optimisation; vGRF, vertical component of ground reaction force; 3D GRF, three-dimensional ground reaction force; EMG-inf, EMG-informed.

## Data Availability

The data supporting the findings presented in this manuscript are available from the corresponding author upon reasonable request.
